# The Origin of Cancer

**Published:** 1902-04-19

**Authors:** D'Arcy Power

**Affiliations:** Senior Surgeon to the Victoria Hospital for Children, Chelsea; Assistant Surgeon to, and Demonstrator of Surgical Pathology at, St. Bartholomew's Hospital, E.C.


### The Origin of Cancer.

By D'Arcy Power, F.R.C.S.Eng., Senior Surgeon to the Victoria Hospital for Children, Chelsea; Assistant Surgeon to, and Demonstrator of Surgical Pathology at, St. Bartholomew's Hospital, E.C.

Whatever be the outcome of the cancer question, there is no doubt that the story of the work which is now being done in connection with this disease will always remain one of the most interesting in the history of medicine, and from time to time it will doubtless be retold in future generations. Never before has so determined an attempt been made to solve the mystery of disease. From the earliest times isolated workers, sometimes separated from each other by centuries, have grappled with similar problems and their efforts have been crowned with greater or less success. But it is only of late years that increased facilities for communication have enabled scientific men in different countries to band themselves together for so definite a purpose as the investigation of the cause of cancer.

### The Cause Unknown.

The distribution of cancer is world-wide and the discovery of its cause therefore is of universal interest. But the cause of cancer is quite unknown at the present time, and any explanation that can be offered is hypothetical, yet the mortality from the disease seems to be increasing and consequently there should be some common cause which ought to be capable of discovery. We are really in no better position to answer the question "What is the cause of Cancer?" than were our ancestors more than two hundred years ago, and many of those now living give an almost identical reply if the allowance be made for the alteration of jargon.

The ablest, the most clear-sighted, and the most practical surgeons of the sixteenth and seventeenth centuries were Ambroise Paré (1510–1590) and Richard Wiseman (1616?–1676). Paré was the Huguenot surgeon to the French kings Charles IX. and Henry III.; Wiseman was serjeant-surgeon to our own king Charles II. Both surgeons wrote of cancer from their own experience and formulated to a large extent their own belief, for neither of them were lovers of authority. Their ideas, therefore, are of greater value to us than those of the physicians, their contemporaries, and this is what they say of cancer.

### Paré's Views.

Paré, using the language of his time, classes cancer with the "tumours against nature in general," or, as we should say, under the heading of pathological new growths. He writes: "A cancer is an hard tumour, rough and unequal, round, immovable, of an ash or livid colour, horrid by reason of the veins on every side, swollen with black blood and spread abroad to the similitude of the stretched out legs and claws of a crab. It is a tumour hard to be known at the first, as that which scarce equals the bigness of a Chick or Cicer (*i.e.*, the chickpea, which is sometimes used in place of coffee); after a little time it will come to the greatness of a hazelnut, unless peradventure provoked by somewhat too acrid medicines it suddenly increase; being grown bigger, according to the measure of the increase, it torments the patient with pricking pain, with acrid heat, the gross blood residing in the veins growing hot, and inferring (producing) a sense like the pricking of needles."

Paré continues in the next chapter, which is headed "Of the Causes of Cancer": "Here we acknowledge two causes of a cancer, the antecedent and conjunct. The antecedent cause depends upon the default of irregular diet, generating and heaping up gross and feculent blood; by the morbific action of the liver disposed to the generation of that blood; by the infirmity or weakness of the spleen in attracting and purging the blood; by the suppression of the courses or hæmorrhoids or any such accustomed evacuation. The conjunct cause is that gross and melancholic humour sticking and shut in the affected part as in a strait. That melancholic blood which is more mild and less malign, only increased by a degree of more fervid heat, breeds a not ulcerated cancer, but the more malign and acrid causes an ulcerated. For so the humour which generates carbuncles, when it hath acquired great heat, acrimony, and malignity, corrodes and ulcerates the part upon which it alights. A cancer is made more fierce and raging by meats inflaming the blood, by perturbations of the mind, anger, heat, and medicines too acrid, oily, and emplastic, unfitly applied both for time and place." Paré is clear and precise about the line of treatment to be adopted in cancer, for he says, "A cancer . . . when it hath once increased admits no cure but by iron (*i.e.* the knife)."

### Wiseman a Century Later.

Wiseman, writing about 1670, or a century later than Paré, says:—"The cause of a cancer is usually said to be adustion of the humours which upon an over-concoction, or rather broiling, grow retorrid and sharp.

"I cannot imagine what heat these authors suppose to be in the body which is capable of making such an adustion (*i.e.*, a dryness) as is here spoken of. I rather impute the corrosive venom that attends this tumour to the materials of which it is made than to any extraordinary heat; and that because we see the highest fever not attended with a cancer, and on the contrary a cancer not often attended with any extremity of heat; so that it cannot be adustion that is the cause of the malady. But I rather think the matter of the humour to be in fault, which by some error in concoction became sharp and corrosive (it may be arsenical, as appears by the sloughs we sometimes find made in a night). This humour, being in itself sharp and corrosive, is apt to convert whatever comes to it of blood into the same acrimony with itself; which is easy to be done by mixing such an acrimonious ferment with a liquor that abounds with acid salts as the blood of such men usually doth. Being such it doth increase apace while the skin is yet whole; but much more when, upon breach of skin, the accession of air adds to the vigour of the ferment upon which it grows fierce and thrusts itself out into fungus, tubercles, etc. The remote cause of this tumour is either a fault in the original constitution of the body, or an acquired one, as by a bruise, tumours, ill-handlings, etc., or it may be an error in diet."

### Treatment in Old Days.

It is clear from these passages that Wiseman was infected with the chemical theory of disease which was prevalent among the physicians of his day. The following extracts show, too, that he was inferior to Paré in his treatment. "The cure of a cancer in general consisteth in these three intentions: First, in the generation of good blood; secondly, in correcting and evacuating of the atrabilious humours in the body; thirdly, in preventing the growth of the tumour and disposing it to discussion (*i.e.*, resolution). . . . If, notwithstanding all your endeavours, the tumour increase and be like to ulcerate, you may do well to forewarn the patient of the danger; and if it be loose and in a place where it may be safely extirpated, propose it to them, lest afterwards they desire it when it is too late.

"That you may be the more successful in the operation I shall offer to your consideration these few qualifications. First, that the patient be of a strong constitution and of a tolerable good habit of body, and not in a declining age when the menstrua are ceased. Secondly, that the cancer be loose and the axilla free from painful glands. It were also to be wished that the cancer took its original from some accident, as a bruise, etc. Thirdly, that the operation be performed in the spring or autumn of the year, lest through the great heat of the summer the spirits be resolved, or by reason of the extreme cold in the winter the native heat should be choked."

### John Hunter.

A hundred years after Wiseman, John Hunter (1728–1793) was laying the foundations of modern surgical pathology. Writing in 1785 he says: "The predisposing causes of cancer are three in number—viz., age, parts, and hereditary disposition; perhaps climate also has considerable effect, though not in itself a predisposing cause. . . . Some suppose cancers to be hereditary, but this I can only admit according to my principle of hereditary right—that is, supposing a person to possess a strong disposition or susceptibility for a particular disease, the children may also; but I have not yet ascertained the generality of this fact. In many persons it would appear that some of the predisposing causes are sufficient to become the immediate ones, as when the diseased action takes place at a certain stated time, without any immediate cause. Are they more frequent in some countries than others from difference of climate? I have heard that they are very rare in the West Indies and in the Friendly Isles, where Captain Cook relates the women fight for prizes in the manner of our prize fighters, and that their blows are chiefly aimed at the breasts. Now, from the medical men not having observed or mentioned a frequency of this disease among the inhabitants of that isle, it would appear not to be so easily produced as in this climate; so most probably climate has some power both in disposing to the disease and in preventing it."

Hunter, however, was not particularly interested in the question of cancer, and he made no experiments to find a cause.

### The increase of Cancer.

The question of the increase of cancer arose at a very much later date, and from time to time it has been actively debated. By many the increase has been accepted as real, whilst to others it has appeared fictitious, in the sense that it could be explained by improved diagnosis, the result of better education on the part of the whole body of the medical profession. The question is now set at rest for England and Wales by the statement of Dr. John F. W. Tatham, who speaks with authority, for he is superintendent of statistics at the General Register Office. He says, "according to the latest returns, the male death-rate from cancer in England and Wales is equal to 672 in each million living, whilst the female death-rate amounts to not less than 975 per million. But this excess is due to the unequal tendency of malignant disease to attack the generative organs of the female rather than of the male, for if the deaths from cancer of the ovaries, uterus and breast are subtracted from the total cancer deaths of females, the remainder gives a death-rate among females which is considerably below that among males. Thus, in the four years 1897–1900, the male death-rate from cancer, less the deaths from disease of the organs above mentioned, correspond to a death-rate of 645 per million, whilst the female rate, with the same limitations, does not exceed 568 per million. The mortality from cancer is not very excessive until some time after the twenty-fifth year of age. Thus, at ages under 35, it averages from 44 to 66 per million, but as age advances the mortality increases in both sexes very rapidly, until at the more advanced ages it is enormous. It is notorious, too, that in recent years the mortality from cancer has increased very rapidly, not only among females, but among males, and although at the present time women suffer much more severely than men from malignant disease, nevertheless, the disease during the last 30 years has increased among men much more rapidly than among women."

### Increase in Germany also.

A similar increase appears to be taking place in Germany, for Dr. Wutzdorff, one of the heads of department in the German Imperial Board of Health has just published a paper containing important statistics about the prevalence of cancer in Germany. His figures deal with (*a*) the statistics of public and private hospitals, and (*b*) with the statistics of the causes of death. The first part cannot be pronounced completely conclusive, since a large percentage of cancer cases are not brought into hospital at all. Still, these figures though incomplete are most striking. It appears that in the year 1879, the number of cancer cases in German hospitals was 6,630 (2,732 male and 3,898 female), whilst in the year 1898 the number had increased to 24,166 (10,100 male and 14,166 female), that is an increase of 266 per cent. And since the total number of hospital patients had doubled (or increased 100 per cent.) in the twenty years, the real meaning of these figures is that—as compared with other diseases—there were more than twice as many cancer cases in 1898 as in 1879. The "cause of death" lists show the increase of cancer from 1892 to 1898. In 1892, 2·6 per cent. deaths were accounted for by new growths, and in 1898, 3·5 deaths per cent. were attributed to the same cause. Of 100,000 inhabitants, 59·6 died of carcinoma in 1892, and 70·6 in 1898—an increase of 18·5 per cent. in six years.

These statements are of very great value and interest. They appear to bear out the contention of those who hold that the increase is to be explained by the greater longevity due to better hygiene associated with greater cleanliness of living amongst the mass of people. But against this is to be placed the statement made by Sir William Mitchell Banks, though confessedly as a general impression only, that "his experience of 35 years points distinctly to the fact that cancer now makes its appearance at a much earlier period of life than it formerly did."

### Modern Theories.

The modern theories of cancer resolve themselves into three great groups, each of which is worthy of a brief consideration :—

1. *The Theory of Spermatic Influence.*—The growth of the tumour is considered on this theory to be due to the spermatic influence of certain cells upon those contiguous to them, the more potent cells stimulating the others to proliferate. The theory is highly speculative and it is bad as a working hypothesis for it does not appear to be capable of much experimental proof. Dr. Beatson has lately revived it in a modified form by showing that in some cases, at any rate, the operation of oöphorectomy in a woman suffering from cancer may lead to a temporary or even permanent diminution in the size of the malignant growth. He holds that the ovaries and testes produce a fructifying fluid elaborated by all the tissues of the body under the stimulus of these organs. The fructifying fluid regulates the power of cell-division within the body which secretes it and it consequently exercises most influence over those cells which have the greatest power of proliferation. When the ovaries and testes dwindle from age, or are removed by the surgeon, the tissues cease to give up this fluid which being retained reacts upon the cells causing them to multiply in an abnormal manner. Cancer, therefore, is the result of this proliferation associated with loss of differentiation, *i.e.* loss of functional activity, so that the cells are no longer of any service to the body and revert practically to the embryonic type. I confess that at present this conveys no more to my mind than does Wiseman's explanation which attributes cancer to an adustion of the humours.

2. *The Theory of Embryonic Residua.*—The theory of "rests" assumes that during the process of intrauterine development more cells may be produced than are actually necessary for the formation of a particular organ or tissue. These superfluous cells may remain dormant for a long time without losing their inherent power of reproduction. Tumours are due to the multiplication of these cells, or to their secreting power as in the case of some cysts. This theory was placed upon a scientific basis by Durante in 1874, and it was promulgated with such ardour in 1875 by Julius Cohnheim (1839–1884), then a talented young professor of morbid anatomy at Breslau, that it has ever since been known as "Cohnheim's theory." Adami and Ribbert have supported and modified the theory within the last few years. It is attractive, and it is partially correct, for some tumours undoubtedly arise in the manner suggested by Cohnheim, and many cysts can be shown to start from embryonic rudiments. But there is no pathological evidence to show that every malignant growth arises in this manner, though there is no doubt that some forms of congenital malformation increase the predisposition to malignant disease.

3. *The Theory of Irritation.*—Irritation as a cause of cancer has been well recognised, at least since the time of Ambroise Paré, and of all the alleged causes, it seems at first sight as if it alone had withstood the lapse of years. But the meaning of the term irritation has varied greatly from time to time. It meant originally direct injury to a part, and it is still used popularly in this sense when a woman says that the scirrhus in her breast came after a blow, the fact being that the injury caused her to examine her breast and thus to find the tumour. Then it was thought that the irritation must be prolonged, and most people still believe that an innocent tumour if irritated sufficiently will become malignant. But there is no evidence to prove that such a transformation of an innocent into a malignant growth really takes place, though it happens from time to time that a malignant tumour may grow near an innocent one, and that a tumour which has increased quietly and slowly for many years may grow rapidly and disseminate. There is nothing however to show that the irritation of the innocent tumour led it to become malignant, and my own experiments of irritating various mucous membranes have wholly failed to produce more than a simple inflammatory condition.

### Micro-organisms.

Within the last few years the irritation theory has assumed a new phase, for the cause has been looked for in connection with micro-organisms of various kinds. This modification of the old doctrine of irritation received a powerful impulse when Sir James Paget said in the course of the first Morton Lecture delivered in 1887 at the Royal College of Surgeons of England : "I believe that micro-parasites, or substances produced by them, will some day be found in essential relation with cancers and cancerous diseases." The micro-parasitic theory was not wholly new, for with the growth of bacteriology many observers had endeavoured to find specific bacteria in malignant growths, but the modification introduced by the veteran pathologist was the suggestion that the micro-organisms might approach rather the animal than the vegetable type, and that it might thus be necessary to adopt new methods of investigation before they could be demonstrated.

Many eager workers at once busied themselves in this branch of research, and the parasitic theory of cancer soon became a well-recognised working hypothesis. The early results were somewhat remarkable. New staining agents were employed, and carefully hardened sections of decadent epithelium were submitted for the first time to a rigid examination under high powers of the microscope. Many unfamiliar appearances were seen, which on the principle *omne ignotum pro magnifico*, were incontinently set down as "cancer parasites" or "cancer bodies." Fortunately, however, most observers made careful drawings of the different structures they labelled in this manner, and it was soon found possible to eliminate most, if not all, by showing them to be altered epithelial cells or various mesoblastic structures which had gained access to the epithelium on one pretext or another. None have survived to the present day without having undergone some essential change, and this, I venture to think, is a strong argument against anyone of them being an essential cause of cancer.

### A Valuable Working Hypothesis.

The irritation theory of cancer remains, in spite of this, the most valuable working hypothesis which has yet been brought forward. It can be submitted to experiment, and it is thus capable of scientific proof or disproof. So far, all experiments have been negative, and the microscopic results have perhaps been the most inconclusive of all. Bacteria, indeterminate bodies probably due to alterations in the tissues from the hardening and staining processes to which they have been subjected; "cancer organisms," yeasts, moulds and other fungi as well as various plasmodia have all had their brief reign, but none have stood the tests required of them, and in no case have they been able to prcduce cancer when they were introduced artificially into an animal's body. The most, therefore, that can be said of any of them is that although they may be often, or even usually, present in cancerous tissues, they are concomitant with, but not causes of, the disease. This was lately shown in a most striking manner by a beautiful specimen exhibited by Dr. Galloway, at a meeting of the Chelsea Clinical Society, in which a non-fungating carcinoma of the skin was supporting a most luxuriant growth of a spore-producing fungus with which it seemed to have become accidentally inoculated.

The parasitic hypothesis seems to have reached its present eminence by the facility with which it lends itself to experimental investigation. But this very ease of investigation has led observers to follow each other like sheep, and has tended to make observation the rule rather than experiment. I have been attempting lately to ascertain whether any of the various appearances described as "cancer bodies" are to found either unchanged or in a modified form apart from cancerous tissues, for if this were possible there might be some hope of identifying and experimenting with some particular type of organism.

### House flies.

For this purpose I submitted flies to a rigorous search, because flies are capable, as is supposed, of carrying various diseases, such as typhoid, cholera, and tubercle, whilst under appropriate conditions they may certainly poison wounds and inflict poisoned wounds. I made myself acquainted with the histology of the London house-fly in autumn and then, by the kindly help of a friend, I obtained specimens of flies from a house where people were ill of cancer and where the disease had occurred with undue frequency. These flies I also submitted to microscopic examination. As I expected, I found no abnormal appearances in the house-flies from either source because these flies have no means of biting or otherwise of inoculating except by what they carry on their feet or mouth parts. But as a result of my inquiries I learnt that two different kinds of fly live together in many parts, the ordinary harmless house-fly and the Stomoxys calcitrans which is armed with lancets capable of biting through the human epidermis and thus able to perform a true inoculation. The fly obtains its name, *calcitrans*, from the fact that it bites cattle on the legs and makes them lash out sideways. It is not easy to distinguish at first sight from the musca domestica or house-fly, and the two are often found together, especially in August and September. The stomoxys, however, holds its head erect when it is at rest, whilst the house-fly stands with its head downwards. It is a little smaller, too, than the house-fly, its wings are broader, and it keeps them unfolded. I have not yet had an opportunity of examining the stomoxys calcitrans, but I am anxious to do so, and I should feel much obliged to anyone who would send me a few living specimens, especially if they were taken from a room where a patient had long been ill of cancer, or from a house where there had been many successive deaths from this disease. The results in all probability will be negative, but in every research a certain amount of unremunerative work has to be done to clear the ground for other observers, and it is well, I think, for those who have but little time at their disposal to assist the general advance by working thoroughly over their own little field.

### Auto-inoculation.

The cases of auto-inoculation are interesting and suggestive, but they argue against, rather than in favour of, the parasitic nature of cancer. The best known instances are when a carcinomatous ulcer spreads to a neighbouring part with which it is usually, but not always, in apposition. Such ulcers have been seen in the bladder, on the labia, in the vagina where a cancerous cervix uteri has rested, and on the skin of the chest when the breast has been the seat of a fungating scirrhus. In 1888 Hahn successfully transplanted several small cancerous grafts from recurrent disease of one breast into the opposite gland, whilst Bergmann and Doyen are said to have repeated his experiment with successful results. There appears to be no room for doubt, therefore, that cancer can be inoculated on persons who already have the disease, the epithelial cells themselves being the infecting agents. Healthy persons, on the other hand, and even those who will subsequently die of cancer, cannot be so inoculated. The few successful cases in man have been those in which a husband with epithelioma of the penis has had a wife with cancer of the uterus. I believe that such examples are mere coincidences and are not the result of infection. In the first place they are very rare, and, like many rarities, they have made an undue impression; secondly, it is becoming increasingly common for a husband and wife to die successively of cancer, usually of different organs and often at considerable intervals of time. The same element of rarity exists in connection with animals, for out of the thousands of inoculations which have been carried out in different parts of the world there have not been more, perhaps, than half a dozen successful results.

### Endemic Cancer.

The endemic character of cancer is worthy of close consideration, and a clue to the origin of the disease may possibly be obtained from an observation of this peculiarity. Mr. Alfred Haviland appears to have been the pioneer in this work, and his results are too well known to require more than brief mention. He drew attention to the fact that cancer occurs with greater frequency in low-lying and water-logged districts than in dry and gravelly soils, and he derived his conclusions almost entirely from statistics. Mr. Shattock, Dr. Law Webb, and some others of my friends soon pointed out that these statistical deductions were confirmed by personal observations.

### Cancer Houses.

They gave examples of houses in which the successive occupants, sometimes of the same family, at other times of wholly different stock, died of cancer, often with appalling frequency and with such certainty that the name "cancer houses" was given to them. Farther investigations by Dr. Nason, Dr. Behla, and others, showed that the cancer houses were often grouped in localities which agreed with the conditions previously laid down by Mr. Haviland. They added, too, that the grouping was so irregular that of two adjoining houses both had not the same baneful reputation. I once had the opportunity of investigating some of these grouped houses, and I found that when the occupants used the same well those in one house might be free from cancer, whilst those in the next house suffered severely. I came to the conclusion therefore that the water supply might be excluded as a cause of cancer.

Considerable interest has been aroused by the statement that cancer is rare where malaria is prevalent, and that African negroes are practically free from cancer. Further investigations, however, show that, like many other statements made about cancer, these observations were based upon incomplete knowledge. Dr. Albert Cook, who has charge jointly with his brother, Dr. J. H. Cook, of the hospital at Mengo, the capital of Uganda, says: "We had over 1,000 in-patients and about 16,000 out-patients under treatment last year . . . among this number were some 1,300 cases of malaria; while cases of malignant disease, though much less common than among Europeans, were by no means rare. . . . Malaria is certainly no bar to the occurrence of carcinoma." It was thought in the same way that the natives of India were exempt from cancer, but this also has been found to be too sweeping a statement; and whilst there is no doubt that cancer is less of a scourge than it is in Europe, it exists to an appreciable extent amongst the native races of the East.

### Jews and Gentiles.

In the same manner it has been repeatedly stated that Jews as a race are less liable to cancer than the peoples amongst whom they dwell, and far-reaching theories as to cancer being due to certain foods have been based upon this alleged peculiarity. Mr. Roger Williams, however, has drawn attention to the statistical tables compiled by Dr. Billings, which prove that the cancer mortality of Jews in the United States is nearly the same as that of the rest of the white population. The cancer mortality per 1,000 for Jews and Gentiles is as follows:—

| | Jews. | Other U.S. Whites. |
|---|---|---|
| Males | ... 13·58 | 13·09 |
| Females | ... 21·65 | 23·59 |

Mr. Roger Williams also points out that Sir Benjamin Ward Richardson, who had considerable experience among the wealthy Jews in the West End of London, found them as prone to cancer as other well-to-do people in the same part of the metropolis.

### Heredity.

The argument derived from heredity against the parasitic theory of cancer no longer has the same weight as it had a few years ago. There is reason to think that the heredity of cancer, like the heredity of tuberculosis, consists in a predisposition towards the disease rather than in a predestination to it as the older physicians thought. It appears in the case of cancer to be some inherited peculiarity in the tissues which leads decadent epithelial cells to run riot under the influence of a stimulus at present unknown. Mr. Roger Williams says that "long study of the family history of cancer patients has convinced me that most of the latter are the surviving members of tuberculous families. . . . Hence I conclude that no hereditary condition is more favourable to the development of cancer than that which gives proclivity to tubercle. The much greater frequency with which obsolete tubercle is found in association with cancer than with most other diseases is also an argument in favour of this view." But in spite of this it is rare to find cancer and tubercle coincidentally in the same person.

Irritation alone is unable to produce a carcinoma, but we have still to find out experimentally whether irritation will produce it in a predisposed animal, and clinically whether irritation and predisposition are always or usually associated. I believe, personally, that there must always be a predisposition which may be hereditary, when it is often well marked, but is more often acquired, for there are many persons suffering from cancer who are quite unable to find any near relatives who have suffered like themselves. In the careful inquiries made by Mr. Roger Williams about the family history of 370 female cancer patients there was a history of heredity in 83, or 22·4 per cent.

### The two Factors.

It is probable, therefore, that there are two factors in the causation of cancer: first, a predisposition, and secondly a stimulus; but the cause of the predisposition is as unknown as the nature of the stimulus. Those who have thought about it most carefully have not advanced a single step beyond Paré, who wrote, "The antecedent cause of cancer depends upon the default of irregular diet generating and heaping up gross and feculent blood." Thus, Sir William Mitchell Banks thinks that the increased predisposition to cancer is associated with the great increase of food, and notably of meat, which is now consumed by civilised people. "The wretched, half-starved patient," he says, "is not the most prone to cancer: its most numerous victims are well-nourished persons with plenty of fat, and often with the colour of health in their cheeks. . . . During the last 20 years the importation of animal food from foreign countries has been enormous. The class of persons who ate meat only once a week 50 years ago no longer exists: vast numbers of persons eat meat daily, and the well-to-do classes eat it three times a day. . . . It is the male who is now beginning to suffer more than the female in proportion to what used to be the case, and it is to be noticed that it is the male who eats the heavy food in ever-increasing quantity, while the female remains much as before in regard to her eating."

### The Question of Diet.

Mr. Roger Williams is also inclined to attribute something to diet, for he says, "long-continued observation of cancer patients in the early stage of the disease has convinced me that most of those affected are large, robust, well-nourished, and florid persons who appear to be overflowing with health and vitality. . . . Those recently attacked never present a cachectic appearance. The small, pale, ill-nourished, and overworked women of the type so familiar in Lancashire and other large industrial centres are seldom the victims of this disease. . . . Statistics show that the consumption of meat has for many years been increasing by leaps and bounds, and it has now reached the total of 131 lbs. per head per year, which is more than double what it was half a century ago. . . . When excessive quantities of such highly stimulating forms of nutriment are ingested by persons whose cellular metabolism is defective, it seems probable that there may thus be excited, in those parts of the body where vital processes are still active, such excessive and disorderly cellular proliferation as may eventuate in cancer. No doubt other factors co-operate, and among these I should be especially inclined to name deficient exercise and probably also deficiency in fresh vegetable food."

### Salt and Cancer.

Dr. Braithwaite is inclined to go still further than this, for he believes that one of the factors leading to the increase of cancer is an "excess of salt in the diet plus a strong local irritant. The excess of salt may arise from individual taste, or from much salt meat, or from too much ordinary meat, which of course involves much salt."

These food speculations are interesting, but they are very difficult either of proof or disproof. I am personally in some doubt whether Sir William Mitchell Banks is correct in thinking that women eat no more than they did 40 years ago, at a time when there was no marked increase in the number of deaths from cancer. In the early days of *Punch* the minute appetites of fine ladies in this country were as perennial a source of fun to the social satirist as has been the conventional mother-in-law at all times and amongst all peoples. To-day, when ladies often surpass in athletic games and in feats of endurance, it is no longer considered indelicate for them to eat heartily and to drink sufficiently. Men, too, are certainly not such gross feeders as they used to be. The Gargantuan feasts and appetites of our ancestors are hopelessly out of fashion and even from the much maligned dinner of a City company the jaded Londoner often comes home refreshed by well-cooked and well-selected food with a moderate allowance of good wine. It may be said with some justice that formerly it was only the few who overate themselves, and then only occasionally, whilst the multitude lived in poverty and all fared more simply than they do at present. But it is precisely amongst the wealthier classes that cancer is increasing, though they now eat more fastidiously than they used to do. I am very doubtful too about the salt hypothesis, for I recall that in the Middle Ages salt entered much more largely into the food of the people than it does at present, for in the Middle Ages there was no winter keep for the cattle; large stocks of provisions were salted each autumn, and fresh meat was not usually eaten again until the spring. The estimation in which lambs' tails are still held as a delicacy, and the dish of lamb on Easter Day are the vestigia of this time and keep in remembrance for us the hankering of our forefathers after fresh meat and the joy with which they welcomed it after the long and unpalatable winter supply of salt provisions had been consumed. Sailors, too, have more than their fair share of salt food, but I have still to learn that they are especially liable to cancer. Tea might as reasonably be set down as a cause of cancer, for its consumption has increased enormously of late years.

In any case the food hypothesis suggests two things in connection with cancer. It may increase the predisposition by causing such an abnormal nutritive activity in the tissues as to lead to excessive proliferation, especially in decadent epithelium, or it may produce the stimulus leading to this proliferation by giving rise to the formation of some chemical irritant at present unknown.

### The Test.

I have often thought that instead of theorising on the truth or falsity of the food hypothesis, in whatever form it is promulgated, it would be very much better to submit it to the test by making a detailed comparison of the ways of living of such classes of the community as retain their original position in regard to cancer. Dr. Law Webb has pointed out that such a people still exist amongst us in some parts of the country, for he says that the colliers of the district in which he practises—Ironbridge in Shropshire—are relatively less liable to cancer than almost any other class. Mr. Roger Williams confirms this statement in the following words: "There are few districts where cancer is less prevalent than in the great colliery centres of Derbyshire, South Wales, Durham, and Lancashire." It should not be difficult, therefore, to ascertain from the old people in these districts whether the meat-eating, salt-eating or tea-drinking habits have changed materially within the last half century. I expect it will be found, as with other people, that the standard of comfort has risen in the usual proportion, and that colliers now eat more meat and less salted provisions than was formerly the case. It is certain that they are infinitely better housed and cared for than when they were practically bondmen, as was the case until the end of the eighteenth century. Some other explanation will then have to be found to account for their comparative immunity. It is interesting to hear in the meantime that those who work in soft coal, as well as those who win hard coal, are less liable to cancer than other persons.

The chemical theory of cancer has not yet passed beyond the speculative stage, but many opportunities of good work appear to exist in connection with it, for, like the parasitic theory, it affords room for experiment.

### A Sea of Doubt.

This outline of the present condition of the cancer question shows that the irritation theory of the origin of cancer now holds sway, though the nature of the irritant is in dispute, and observers are not even agreed whether it is the predisposing or the exciting cause of the disease. It is not known whether the irritant is animal, vegetable, or chemical in its nature; whether it is formed within the body or gains access from without. The irritant hypothesis is good for working purposes as it can be submitted to the test of experiment, and we may hope that in the near future the method of exclusion will give us some firm basis of fact which will in due course enable us to arrive at some definite conclusion about the origin of this formidable disease.

